# Mass Spectrometry for Biomedical and Food Analysis

**DOI:** 10.3390/molecules29061290

**Published:** 2024-03-14

**Authors:** Xianjiang Li, Wen Ma

**Affiliations:** 1 Key Laboratory of Chemical Metrology and Applications on Nutrition and Health for State Market Regulation, Division of Metrology in Chemistry, National Institute of Metrology, Beijing 100029, China; 2 State Key Laboratory of Natural and Biomimetic Drugs, School of Pharmaceutical Sciences, Peking University, Beijing 100191, China

## 1. Introduction

Biomedical and food analysis has always been an important topic that closely relates to health. The sensitive and accurate detection of related components (metabolites, proteins/peptides, drugs, nutrient contents, pesticides, and so on) are critical in understanding the molecular mechanism of biological functions and guaranteeing food safety and people’s health [1]. However, the inevitable complexity of the practical matrix has encouraged researchers to continuously exert significant effort in developing innovative methods with accuracy, rapidity, and simplicity [2]. Relying on its high accuracy, great sensitivity and versatility, and reliable qualitative and quantitative ability, mass spectrometry-related technology is perfect for biomedical and food analysis [3]. On the other hand, sample preparation plays an essential role in analyte enrichment and interference removal, further increasing specificity and sensitivity [4]. Moreover, certified reference materials are essential to guarantee reliable and comparable results [5].

This Special Issue gathers the latest research trends in biomedical and food analysis. By collecting the contributions of specialists in the field, this Special Issue aims to advance knowledge and increase the expertise on biomedical analysis, pharmaceutical science, and food chemistry.

## 2. Summary of Published Articles

This Special Issue includes twelve manuscripts which address the latest advances, including five on biomedical research and seven on food analysis.

Nature is a bank of active compounds among which humans have been seeking drugs [6]. *Hyssopus cuspidatus* Boriss is a traditional Uyghur medicine for the treatment of asthma. Dr. Tie (contribution 1) demystified its molecular mechanism and revealed its pharmacology with an asthmatic mice model. Rosmarinic acid (RosA), the main active constituent, could regulate cytokine levels and inhibit the signaling pathway to suppress inflammation, such as TNF-α and IL-4. Additionally, the animal ethology score and lung histology analysis confirmed its potency. Moreover, some other constituents neutralized the side reaction caused by RosA. Further in-depth research is needed to make RosA a treatment for asthma in humans. Together with medical treatment, early diagnosis is very helpful. Therefore, novel biomarker discovery is a very meaningful topic, especially differentially expressed genes/proteins of cancers. Neagu’s group (contribution 2) used 2D-PAGE-nLC-MS/MS to identify the differences in tumorigenic pathways with MCF7 breast cancer cells that were silenced with the human jumping translocation breakpoint. Their results demonstrated the tumor-suppressive function of this gene that might be a tumor biomarker for breast cancer. Related signaling and metabolic pathways included EMT, ERK/MAPK, PI3K/AKT, Wnt/β-catenin, mTOR, C-MYC, NF-κB, IFN-γ and IFN-α responses, UPR, and glycolysis/gluconeogenesis. Accordingly, LC-MS-based omics is a powerful tool [7,8], especially for disease diagnosis. Metabolites can reflect many pathological or internal changes in biochemical pathways at the molecular level [9]. Dr. Kośliński (contribution 3) used them to develop an amino acid metabolic profile method for glioblastoma and meningioma diagnosis. From the ROV curve results, four targets (lysine, histidine, α-aminoadipic acid, and phenylalanine) demonstrated statistically significant differences, especially phenylalanine. Furthermore, cysteine turned out to be the most important in the decision-making algorithm of the classification tree. These results indicate the importance of mass spectrometry in marker discovery. Compared to LC-MS, matrix-assisted laser desorption/ionization (MALDI) has major advantages in time and throughput. Antonio Monopoli and colleagues (contribution 4) analyzed cyclic tetrapyrroles in blood, bovine liver, fish liver, and mussel samples with different matrixes. In detail, α-cyano-4-chlorocinnamic acid was more effective than proton transfer and electron transfer matrices for protoporphyrin analysis, whereas α-cyano-4-hydroxycinnamic acid facilitated the better detection of heme b and c. This work paved the way for better understanding of the cyclic tetrapyrroles in biological samples. Dr. Liu (contribution 5) comprehensively summarized the recent progress in major depressive disorder research from four dimensions, including analytical platforms, strategies, key metabolites, and antidepressant treatment. In this manuscript, key metabolic challenges are described, and current challenges and prospects are discussed. She hopes this work will stimulate further advances in the field of major depressive disorder research with MS-based metabolomics.

Certified reference material (CRM) is very important in guaranteeing the reliability of detection [10]. Especially, pure CRM is located at the top of the metrological traceability chain [11]. Dr. Ma (contribution 6) developed a vinyl acetate CRM (GBW (E) 062710) that is a restricted substance in food products. For pure CRM development, the identification and quantification of structurally related impurities represent a large bottleneck [12,13]. Three structurally related impurities were elucidated and their correction factors were calculated in the vinyl acetate CRM. Finally, its purity was assigned using the mass balance method with a value of 99.90% and expanded uncertainty of 0.30%. Similarly, Dr. Li (contribution 7) carried out purity assessment of dinotefuran, which is a widely used pesticide. Eventually, the DNT CRM was assigned a mass fraction of 995 mg/g with both mass balance and quantitative nuclear magnetic resonance spectroscopy. During real sample analysis, matrix components affect the accuracy of the final results. Thus, matrix CRMs are very important to realize consensus values. Mr. Li and coworkers (contribution 8) used both HPLC-ICP-MS and HPLC-ESI-MS/MS methods to certificate selenium-enriched yeast reference material. Both methods yielded consistent results for the selenium supplement and the certified value of selenomethionine was 716 mg/kg with expanded uncertainties of 36 mg/kg.

Pesticide residue is a tough problem in food safety, especially during simultaneous analysis of hundreds of them [14,15]. Dr. Zou (contribution 9) proposed an m-PFC method with an SBA-15-C_18_ sorbent. This method was reliable for 139 pesticides. Moreover, 314 samples of nine types of fruits and vegetables were analyzed to assess dietary intake risk. In detail, procymidone most often exceeded the MRLs and tebuconazole was most frequently found. Organophosphorus flame retardants (OPFRs) are another common residue in foods. Due to their large numbers of variants, the high-resolution mass spectrometry-based screening method is very popular. One important prerequisite for novel OPFR discovery is the elucidation of their fragmentation pathway. Dr. Li with her team (contribution 10) found that alkyl and halogenated OPFRs underwent three McLafferty hydrogen rearrangements and aromatic OPFRs cleaved not only the C-O bond but also the P-O bond. Moreover, the substituents had a large effect on the cleavage, as shown by the figures. These fragmentation laws can provide great help in screening OPFR pollutants.

In addition to hazardous residue, two manuscripts focused on nutritional components. Dr. Li (contribution 11) investigated the ginsenoside profile in steamed *Panax quinquefolius* using UPLC-Q-TOF-MS. In total, 175 ginsenosides were identified, and 10 new ginsenosides, and 3 new aglycones were discovered. They also mentioned that the steaming temperature had a larger influence than time on the chemical components after principal component analysis. Some typical position changes and configuration inversion were elucidated simultaneously. The most abundant protein in milk is β-casein, and A1 is one variant that has a histidine moiety in the 67th position. A1 β-casein is harmful for humans; therefore, high specificity is very important in distinguishing all variants. Dr. Li (contribution 12) reported a simple thermolytic digested method to produce characteristic peptides without any denaturing reagents. Thermolysin was chosen for digestion at 60 °C for 4 h. These shorter thermolytic peptides with 11 or 18 amino acid moieties are more suitable for LC-MS analysis than tryptic characteristic peptides with 49 amino acid moieties. This method was successfully applied to commercial milk samples.

## 3. Conclusions

The twelve articles published in this Special Issue cover the latest advances in mass spectrometry for biomedical and food analysis. As we can see, high-resolution omics is very useful in finding biomarkers and revealing their mechanism. Moreover, novel CRM development is a trend in dealing with new contaminants in food analysis. We hope these Special Issues tracking the recent progress will provide beneficial information to readers.
